# A Treatise on Gonorrhœa and Syphilis

**Published:** 1859-11

**Authors:** 


ART. III.—*A Treatise on Gonorrhœa and Syphilis.* By SILAS DURKEE, M.D., etc., etc. With Eight Colored Plates. 8vo., pp. 442. Boston: John P. Jewett & Co., 1859.

*A Treatise on the Venereal Disease.* By JOHN HUNTER, F.R.S. With copious Additions by DR. PHILIP RICORD, Surgeon of the Hôpital du Midi, Paris, etc. Translated and edited, with Notes, by FREEMAN J.

Bumstead, M.D., etc. Second edition, revised, containing a Résumé of Ricord's recent Lectures on Chancre. 8vo., pp. 552. Philadelphia: Blanchard & Lea, 1859.

The former of these volumes is the first attempt at an altogether original work on venereal diseases which has ever been made in this country. Few American practitioners have opportunities for observation, in this department, so ample as to warrant them in claiming a seat among authoritative syphilographers. Dr. Durkee, however, lays before the profession the results of his experience, making it his paramount object to furnish a practically useful book. How far he has succeeded in performing this task satisfactorily we have now to examine.

Common usage has given a significance to the word *gonorrhœa*, which is acknowledged by our author in his adoption of it upon the title-page of his book; but his first chapter is upon *blennorrhagia*, and this latter word seems to be used by him very much more frequently than the former. Neither term is strictly correct, but we decidedly prefer the less pedantic, and, to most minds, more suggestive one first mentioned.

With regard to the cause of gonorrhœa, we must express our skepticism concerning those unfortunate cases where suppurative urethritis is said to occur without any specific influence. So many persons of our acquaintance have eaten salt meat, been intemperate, danced to excess, had rheumatism, and ridden hard-trotting horses, without this result, that it really seems as if another element must have been unacknowledged in the few cases where it has been ascribed to these causes alone. Nor can we believe that a healthy male urethra is thus affected by contact with a simple leucorrhœal discharge, acid or alkaline. Dr. Durkee has weakened the position he takes upon this latter point at page 6, by his admissions at page 158. But, in fact, the perfidy of Venus has such a universally acknowledged effect upon her victims, that actual observation is indispensable to the establishment of any opinion concerning these matters. Perhaps no surgeon ought to be convinced of the possibility of the cases alluded to, unless by experience in his own person.

The treatment of gonorrhœa is the chief topic of Dr. Durkee's second chapter. We agree with him especially in condemning the use of strong injections into the urethra, and in awarding to copaiba and cubebs a value which it is at present the custom of some surgeons to deny them. Here, however, we must object to the use of such terms as "anti-gonorrhœal powers," "a sedative and anti-blennorrhagic influence;" they are not in accordance with sound therapeutics, and might readily mislead the inexperienced.

At the end of this chapter, in connection with the subject of urethral hemorrhage, an anecdote is related, which we think unnecessary and indecent. On page 113, also, is a very diffuse statement of a case, open to the same remark; and throughout the book there are passages which, although generally much less offensive than those just mentioned, are suggestive of another style of literature. Prudery is impure; but there is a certain propriety which can and must be maintained in dealing with these subjects, and which is especially desirable as the distinctive mark of those who study them seriously. Let us not be thought to accuse our author of any impurity in thought; but as we would handle a chancre or a bubo with circumspection, so must we think and speak of this vilest self-inflicted infirmity of our race. Medical men see too much of the fearful issue of these diseases, to make them the subject of trifling remark.

In the chapter on balanitis, our author discountenances slitting up the prepuce when chancres exist within it, and it is in a state of phymosis, lest the wound so made should assume a syphilitic character. We are, however, convinced that, unless there is a free entrance for our local applications, and a free exit for the discharge, such a course is the proper one. Absorption is not more to be dreaded from a large than from a small extent of surface, while a great advantage is gained in laying open the seat of disease to inspection and treatment. The deformity left is but trifling, and the lips of the incision are under control from the outset.

Dr. Durkee assigns less value to the practice of compression in swelled testicle than it seems to us to merit. We have often found that it actually diminished the tenderness of the part, and are inclined to believe that less induration is apt to remain in those cases where it has been employed. Another plan which sometimes answers, and which is particularly useful as a preparative to compression when the tenderness is extreme, is the application of astringent and anodyne dressings, such as lint soaked in lead-water and laudanum.

The chapters introduced in this portion of the work on herpes, eczema, and excoriations of the prepuce, would have been more appropriately placed in connection with the subject of syphilis; it may also be a matter of doubt whether those affections required the allotment of so much space. Nor does it seem to us that spermatorrhœa could be included in a strict construction of the title adopted by our author, especially as he has altogether omitted to speak of strictures of the urethra, which are, in so large a majority of cases, directly traceable to gonorrhœa.

We now come to the portion of Dr. Durkee's work which has reference to syphilis. And here there is a strange jumbling-up of subjects, which seriously impairs any value that may be claimed for the text. What a chancre really is, and how it is to be recognized, is nowhere clearly stated; we read of cartilaginous, Hunterian, inflammatory, and phagedenic chancres, of the views of authors concerning various points, but on seeking for a satisfactory explanation of these terms, we are disappointed. Nor do we think that it is well to use such terms, as denoting different varieties of chancre.

A syphilitic ulcer, a primary sore, has the peculiarity of inducing a sore like itself wherever its matter is applied to a raw surface. It may also give rise, after a time, to certain general secondary and tertiary symptoms; although this property is by many ascribed only to the indurated form.

Now, either a soft or an indurated chancre may take on a phagedenic form, or become surrounded by inflammation; its chancrous character is retained, the new phenomena being simply superadded to the old. These conditions do not, therefore, constitute varieties, strictly speaking, and confusion arises from regarding them in that light. Such at least is the view which we hold, although, for the mere sake of convenience, the descriptions of these forms may be very properly separated. It is because a distinction is drawn between the accidentals as if they were essentials that the student is led to imagine several mysteries, instead of only one.

We do not believe, with Dr. Durkee, that there is a "duality of chancrous virus." We believe that any of the above-mentioned forms may, by inoculation, produce any other, according to certain conditions residing in the part or the individual inoculated; and that all are followed by the same secondary and tertiary phenomena. Nor is there, in our opinion, any difference between a chancre and a common sore, except in the inexplicable communicability of the former, and in the sequelæ of which other parts become the theatre.

As to the *masked* chancre of our author—*concealed* is a much better word, *urethral* is better still—it is of no importance, except as regards its diagnosis and some difficulties in its treatment. But upon this latter point we are somewhat surprised to find that the local applications recommended are so much less powerful than those for the external sore. It seems as if the nitrate of silver, applied by means of a porte-caustique, would afford a much greater degree of security than any other remedy available under the circumstances. To use nitric acid or potassa fusa for an external chancre, and to content ourselves with chloride of soda in solution for a urethral one, would imply that the latter was a source of less serious apprehension.

Dr. Durkee speaks very strongly in favor of the potassio-tartrate of iron, both as a general and as a local remedy, in cases of phagedenic chancre; he recommends also the use of carbonate of ammonia internally. Having never employed the former preparation, we cannot speak from experience in regard to it; but we have been very well satisfied with the effects of very dilute nitric acid as a wash, and chloride of zinc or soda as a steady dressing, in these cases; while the permanent stimulants seem to us, as a general thing, to accomplish more than the diffusible.

Our author advocates the use of small blisters, with a subsequent dressing of mercurial ointment and muriate of ammonia, in the treatment of buboes; but he very properly states that no plan can be implicitly relied on either for their prevention or for their cure. His favorite application, in cases where an indolent ulcer is left after the opening of a bubo, is the potassio-tartrate of iron; we have sometimes found a paste made with chloride of zinc, ʒss to ℥j of starch, to have an excellent cleansing and stimulating effect in this condition of things.

It would extend this review to an unwarrantable length, should we go into an analysis of the remaining chapters of Dr. Durkee's work, embracing secondary and tertiary syphilis; a few points only can be touched upon.

The interesting question, whether secondary syphilis can be communicated to an individual without his or her ever having a primary sore of the usual kind, is discussed in a separate chapter. Dr. Durkee is inclined to believe that such a thing may happen; he cites five cases of his own, and quotes others from different writers, in proof of this view.

There are, however, some considerations which should be taken into the account in weighing this question. In the first place, the cases are but few which support the idea at all. The patients are nearly all women, in whom it is, of course, more difficult to ascertain either the existence or the non-existence, past or present, of a chancre. And of those of the other sex, perhaps not one would imagine that he could have a venereal sore anywhere but on the penis; as to the children in whom the disease is hereditary, they cannot be said to receive it—it is a part of them. Hence we would place this matter in the same category with one or two before mentioned; we confess that our skepticism could only be removed by experience in our own person.

The secondary and tertiary symptoms of syphilis receive an extended notice from our author. We are not altogether satisfied with the classification given by him of the syphilodermata; at least it would have been more valuable to the inexperienced if some explanatory comments had been added. Nor are the pictures, in our opinion, at all illustrative; they can be of no use either to the inexperienced or to those who are able to note their defects.

We must remark that Dr. Durkee's style is often unpleasantly stilted. "The testicular organs and their corporeal forces," "the buccal cavity," "the vascular and dermoid tissues," "cuticular discharges," are not only awkward, but incorrect expressions; they are specimens of a certain florid redundancy to which the author seems quite attached, but which weakens very seriously the force of his reasoning.

It would have been well had references been given more generally where opinions and passages are quoted, as there are many readers who may desire to look them up in their originals. No matter of convenience is more commonly neglected than this, which is often really of importance to the reader and to the author quoted; to the latter it is perhaps a right which should always be conceded. And here let us claim for the quoted another of their dues, which is, that their names should be spelt correctly. On page 193 of the present volume we find "Boek" and "Danielson" for "Boeck" and "Danielssen." Foreign authors do indeed mutilate English and American names, but with an honest awkwardness which shows that it is their misfortune and not their fault.

In making the foregoing comments upon Dr. Durkee's work, we have not been prompted by a fault-finding spirit. We consider that authors sustain a certain obligation to uphold the credit of their country and of their profession; if their productions fall short of this, the reviewer has his duty also to perform, without fear or favor. Should his judgment be erroneous, it must and will be set aside by that of the profession at large.

The work of Hunter, with the additions of Sir E. Home, and the notes of Babington and Ricord, maintains a most enviable position among monographs. It passes now for the second time through the hands of Dr. Bumstead, who has added extracts from M. Ricord's recent lectures, as reported by M. Alfred Fournier.* As for the older portions of this valuable treatise, they have so long stood the test of professional criticism that it would be superfluous for us to discuss them anew; we have only to speak of that which is now for the first time presented in an English dress. M. Fournier's reports have already been criticised in this Review, (July, 1858,) so that in fact it is the dress itself with which we are now concerned.

Dr. Bumstead has, in our opinion, succeeded very well in embodying the views held at the Hôpital du Midi in his notes to the present edition. These views, in regard to the difference between simple and infecting chancres, are contained in a note nearly ten pages in length, substituted for the latter portion of Ricord's original note to Hunter's chapter on chancre. Appended to the chapter on the constitutional treatment of chancre, is a note setting forth the therapeutical indications deduced from those views; and some new ideas concerning buboes are to be found in a

* Leçons sur le Chancre. Professées par le docteur Ricord; redigées et publiées par Alfred Fournier, Interne de l'Hôpital du Midi. Paris, 1858.

note substituted for that formerly placed in connection with the chapter on buboes in women.

On page 497 we find a note inserted, in which mercury is virtually called a specific for the earlier constitutional manifestations of syphilis, and iodide of potassium for the tertiary. Perhaps there are very many surgeons who would disclaim this idea theoretically, who, nevertheless, act upon it daily in their practice.

The employment of the chlorate of potassa as a preventive of the disagreeable effects of mercury, is alluded to with favor by Dr. Bumstead, in a note on page 502.

M. Ricord is what is termed, in American popular parlance, a "smart" man. His writings are not so much clear and convincing by their earnestness and perspicuity, as they are brilliant and startling; so that one is dazzled and carried away by his meretricious casuistry, rather than compelled to yield to the force of his arguments. Not that we would deny his correctness, perhaps, in any of his positions; but we feel that we are in the hands of a dexterous reasoner, who could prove one side of a question as well as the other, if he chose to do so. Hence we have always, we must confess, when reading his arguments, somewhat the feeling with which one watches a juggler; his results are arrived at often with a startling suddenness, and stated with an easy flippancy which would be apt to overmatch the opposition of an ordinary plodder in medical reasoning.

His doctrine as to the simple and the infecting chancre, developed as it now is, seems to us to involve the advocates of syphilization in a dilemma. For if a man who has had one indurated chancre will not have another, then the giving of it to him simply converts a mere risk into a certainty. If soft chancres only are caused by artificial inoculation, how many soft chancres are equivalent to one of the indurated variety, against which alone it is so desirable to obtain immunity?

In the twenty-third of his "Letters on Syphilis," M. Ricord somewhat pathetically exclaims: "Oh, Syphilis, when will you be understood?" And when we remember that it is not many years since a work was issued on the non-existence of a syphilitic virus—that it is at present undecided whether there be one virus or two*—when we look at the theory, certainly not one to be carelessly rejected, of syphilization, we can but ask, does the mystery that envelops so many parts of this subject reside in the facts themselves, or in the minds of those who study them? Are we any nearer to the true pathology of venereal diseases than the surgeons of the last century were, or have we only gained so much caution that we do less mischief in the way of medication than they did?

---

* Ricord, Leçons sur le Chancre, p. 227.

